# New Study Reveals Long-Term Effects of MDMA on the Brain’s Glutamate-Glutamine Complex

**DOI:** 10.1093/ijnp/pyad048

**Published:** 2023-08-07

**Authors:** Apochi Obed Okwoli

**Affiliations:** Neuropsychology, School of Behavioral Science, National Forensic Sciences University, Gandhinagar, Gujarat, India

Dear Editor,

I am writing to discuss the significant findings presented in the study titled “Chronic 3,4-Methylenedioxymethamphetamine (MDMA) Use Is Related to Glutamate and GABA Concentrations in the Striatum But Not the Anterior Cingulate Cortex” (Zimmermann et al., 2023). The research conducted by Zimmermann and colleagues provides valuable insights into the long-term effects of MDMA use on the brain, particularly in relation to the glutamate-glutamine complex (GLX) levels in the striatum.

The study’s findings shed light on the impact of chronic MDMA use on the principal excitatory neurotransmitter system in the brain in addition to its well-known effects on the serotonin system. MDMA, commonly known as ecstasy, is a widely used recreational drug known for its empathogenic and euphoric effects. However, there has been growing concern about the potential long-term consequences of MDMA use on brain function and mental health (Gill et al., 2020; De Gregorio et al., 2021).

The discovery of alterations in GLX levels in the striatum suggests that MDMA use may have broader neurobiological effects than previously believed (Mustafa et al., 2020). Glutamate and GABA are the primary excitatory and inhibitory neurotransmitters in the central nervous system, respectively, and their balance is crucial for maintaining normal brain function. Disruptions in this delicate balance have been implicated in various neurological and psychiatric disorders (Sood et al., 2021).

The study’s findings indicate that chronic MDMA use is associated with increased GLX levels in the striatum (Zimmermann et al., 2023). This increase in glutamate and glutamine concentrations suggests enhanced excitatory activity in this brain region. Such alterations in GLX levels may contribute to the cognitive deficits often observed in MDMA users, such as impaired impulse control and declarative memory (Suarez et al., 2002). The striatum, a key component of the basal ganglia, is involved in motor control, reward processing, and habit formation. The increased GLX levels in the striatum of MDMA users may disrupt the balance in the fronto-striatal pathways associated with impulsive behavior (Gysling et al., 2020).

Understanding the long-term effects of MDMA on the brain is crucial for developing effective prevention and intervention strategies. MDMA use is particularly prevalent among young adults who engage in recreational activities such as music festivals and clubs. By elucidating the specific neurochemical changes associated with chronic MDMA use, this study provides valuable insights into the underlying mechanisms that contribute to the cognitive impairments observed in MDMA users.

It is important to note that this study focuses on the striatum and does not find significant alterations in GLX levels in the anterior cingulate cortex (ACC) (Zimmermann et al., 2023). The ACC plays a critical role in various cognitive and emotional processes, including decision-making, attention, and error detection. The lack of changes in GLX levels in the ACC suggests that the effects of chronic MDMA use may be region specific. This finding highlights the need for further investigation into the differential effects of MDMA on various brain regions to gain a comprehensive understanding of its neurobiological consequences.

The implications of these findings extend beyond the scientific community. Public health initiatives aimed at raising awareness about the potential risks associated with MDMA use can benefit from incorporating this research. Education campaigns targeted at young adults, health-care professionals, and policymakers should emphasize the long-term cognitive consequences of MDMA use. By providing evidence-based information, these initiatives can empower individuals to make informed decisions and promote safer recreational practices.

Moreover, the authors’ rigorous research and valuable contribution to the field deserve commendation, from well-defined inclusion and exclusion criteria to careful screening of the MRS data to exclude participants artifacts. The researchers also used a robust regression model to confirm group differences and minimize the influence of outlier data points. Additionally, the study used correlation analysis to assess the relationship between metabolite concentration and frequency of MDMA use among drug users (Zimmermann et al., 2023). Conducting research on illicit substances such as MDMA poses inherent challenges due to legal and ethical considerations. The study’s findings advance our understanding of the neurobiological effects of chronic MDMA use and provide a foundation for future investigations.

The findings presented in this study have the potential to inform future studies investigating the neurobiological effects of MDMA and may contribute to the development of targeted interventions to mitigate the cognitive deficits associated with MDMA use. For instance, cognitive training programs tailored to address specific cognitive impairments observed in MDMA users may help improve cognitive function and quality of life in this population.

In conclusion, the study titled “Chronic 3,4-Methylenedioxymethamphetamine (MDMA) Use Is Related to Glutamate and GABA Concentrations in the Striatum But Not the Anterior Cingulate Cortex” (Zimmermann et al., 2023) provides important insights into the long-term effects of MDMA use on the brain. The observed alterations in GLX levels in the striatum suggest broader neurobiological effects of chronic MDMA use, which may contribute to cognitive deficits commonly observed in MDMA users. Understanding the neurochemical changes associated with MDMA use is essential for developing effective prevention and intervention strategies. I commend the authors for their valuable contribution to the field, and I believe that the discussion of these significant findings will contribute to the ongoing dialogue surrounding the long-term effects of MDMA on the brain.

Thank you for your time and consideration.

Sincerely,

Apochi Obed Okwoli
